# (2*E*)-1-(3-Chloro­phen­yl)-3-(4-chloro­phen­yl)prop-2-en-1-one

**DOI:** 10.1107/S1600536809037805

**Published:** 2009-10-03

**Authors:** Jerry P. Jasinski, Ray J. Butcher, B. Narayana, K. Veena, H. S. Yathirajan

**Affiliations:** a Department of Chemistry, Keene State College, 229 Main Street, Keene, NH 03435-2001, USA; b Department of Chemistry, Howard University, 525 College Street NW, Washington, DC 20059, USA; c Department of Studies in Chemistry, Mangalore University, Manalaganotri 574 199, India; d Department of Studies in Chemistry, University of Mysore, Manasagangotri, Mysore 570 006, India

## Abstract

The title compound, C_15_H_10_Cl_2_O, is a chalcone with 3-chloro­phenyl and 4-chloro­phenyl substituents bonded at the opposite ends of a propenone group, the biologically active region. The dihedral angle between mean planes of these two chloro-substituted benzene rings is 46.7 (7)° compared to 46.0 (1) and 32.4 (1)° in similar published sructures. The angles between the mean plane of the prop-2-en-1-one group and the mean planes of the 3-chloro­phenyl and 4-chloro­phenyl rings are 24.1 (2) and 29.63°, respectively. While no classical hydrogen bonds are present, weak inter­molecular C—H⋯π-ring inter­actions are observed, which contribute to the stability of crystal packing.

## Related literature

For the potential use of chalcones or chalcone-rich plant extracts as drugs or food preservatives, see: Dhar (1981 ▶). For the biological and pharmaceutical activity of chalcones, see: Dimmock *et al.* (1999 ▶); Troeberg *et al.* (2000 ▶); Ram *et al.* (2000 ▶). For their applications as organic nonlinear optical materials, see: Sarojini *et al.* (2006 ▶). For the bis-(4-chloro­phen­yl) analog, see: Wang *et al.* (2005 ▶) and for the (2-chloro­phenyl, 4-chloro­phen­yl) analog, see: Fun *et al.* (2008*b* ▶). For antitumor and antioxidant activity studies and non-linear optical studies, see: Mukherjee *et al.* (2001 ▶); Poornesh *et al.* (2009 ▶); Shettigar *et al.* (2006 ▶, 2008 ▶); Wang *et al.* (1997 ▶). For related structures, see: Butcher *et al.* (2007 ▶); Fischer *et al.* (2007 ▶); Fun *et al.* (2008*a* ▶); Harrison *et al.* (2006 ▶); Ng *et al.* (2006 ▶); Teh *et al.* (2007 ▶); Yathirajan *et al.* (2006 ▶).
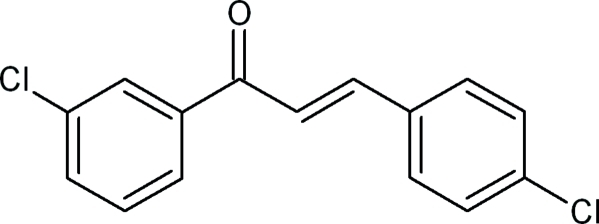


## Experimental

### 

#### Crystal data

C_15_H_10_Cl_2_O
*M* *_r_* = 277.13
Triclinic, 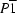

*a* = 5.8884 (9) Å
*b* = 7.3328 (9) Å
*c* = 14.6752 (16) Å
α = 102.821 (10)°
β = 95.003 (10)°
γ = 92.933 (11)°
*V* = 613.88 (14) Å^3^
*Z* = 2
Cu *K*α radiation
μ = 4.61 mm^−1^
*T* = 110 K
0.53 × 0.33 × 0.28 mm

#### Data collection

Oxford Diffraction Gemini R CCD diffractometer
Absorption correction: multi-scan (*CrysAlisPro*; Oxford Diffraction, 2007 ▶) *T*_min_ = 0.067, *T*_max_ = 0.275
4041 measured reflections
2402 independent reflections
2147 reflections with *I* > 2σ(*I*)
*R*_int_ = 0.035

#### Refinement

*R*[*F* ^2^ > 2σ(*F* ^2^)] = 0.047
*wR*(*F* ^2^) = 0.133
*S* = 1.04
2402 reflections
163 parameters
H-atom parameters constrained
Δρ_max_ = 0.48 e Å^−3^
Δρ_min_ = −0.39 e Å^−3^

### 

Data collection: *CrysAlis Pro* (Oxford Diffraction, 2007 ▶); cell refinement: *CrysAlis RED* (Oxford Diffraction, 2007 ▶); data reduction: *CrysAlis RED*; program(s) used to solve structure: *SHELXS97* (Sheldrick, 2008 ▶); program(s) used to refine structure: *SHELXL97* (Sheldrick, 2008 ▶); molecular graphics: *SHELXTL* (Sheldrick, 2008 ▶); software used to prepare material for publication: *SHELXTL*.

## Supplementary Material

Crystal structure: contains datablocks global, I. DOI: 10.1107/S1600536809037805/zs2009sup1.cif

Structure factors: contains datablocks I. DOI: 10.1107/S1600536809037805/zs2009Isup2.hkl

Additional supplementary materials:  crystallographic information; 3D view; checkCIF report

## Figures and Tables

**Table 1:** Hydrogen-bond geometry (Å, °)

*D*—H⋯*A*	*D*—H	H⋯*A*	*D*⋯*A*	*D*—H⋯*A*
C2—H2*A*⋯*Cg*2^i^	0.95	2.98	3.608 (2)	125
C5—H5*A*⋯*Cg*2^ii^	0.95	2.88	3.488 (2)	126
C14—H14*A*⋯*Cg*1^iii^	0.95	2.77	3.474 (2)	131

Symmetry codes: (i) 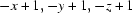; (ii) 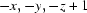; (iii) 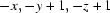. *Cg*1 is the centroid of the C1–C6 ring and *Cg*2 is the centroid of the C10–C15 ring.

**Fig. 1.**
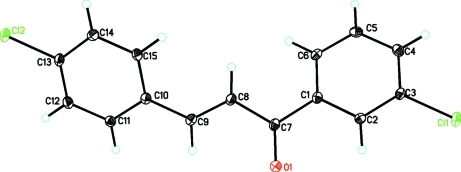
Molecular structure of the title compound (I) showing the atom labeling scheme and 50% probability displacement ellipsoids.

**Fig. 2.**
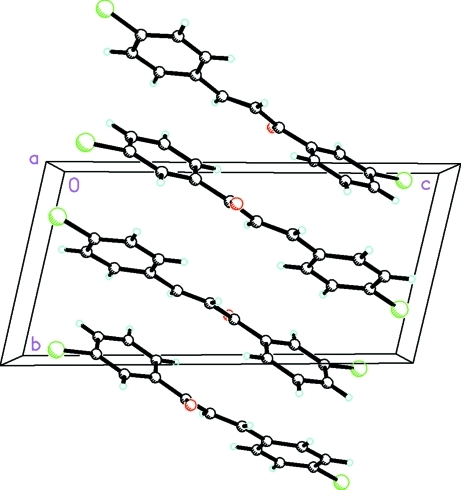
Packing diagram of the title compound viewed down the a axis of the unit cell.

## supplementary crystallographic information

### Comment

Chalcones or 1,3-diaryl-2-propen-1-ones, belong to the flavonoid family. Chemically, they consist of open-chain flavonoids in which the two aromatic rings are joined by a three-carbon α, β-unsaturated carbonyl system. A vast number of naturally occurring chalcones are polyhydroxylated in the aryl rings. The radical quenching properties of the phenolic groups present in many chalcones have raised interest in using the compounds or chalcone-rich plant extracts as drugs or food preservatives (Dhar, 1981). Among the many useful properties that chalcones have been reported to possess include anti-inflammatory, antimicrobial, antifungal, antioxidant, cytotoxic and anticancer activities (Dimmock *et al.*, 1999). Many chalcones have been assessed for their high antimalarial activity, which is probably a result of Michael addition of nucleophilic species to the double bond of the enone (Troeberg *et al.*, 2000; Ram *et al.*, 2000). Chalcones are also finding applications as organic non-linear optical (NLO) materials due to their good SHG conversion efficiencies (Sarojini *et al.*, 2006). Owing to the importance of these flavanoid analogs, the title chalcone (I), C_15_H_10_Cl_2_O has been synthesized and its crystal structure is reported here.

The title compound is a chalcone with 3-chlorophenyl and 4-chlorophenyl rings bonded at the opposite ends of a propenone group which is the biologically active region. The dihedral angle between mean planes of these two chloro-substituted benzene rings is 46.7 (7)° compared to 46.0 (1)° in the bis-(4-chlorophenyl) analog (Wang *et al.*, 2005) and 32.4 (1)° in the (2-chlorophenyl, 4-chlorophenyl) analog (Fun *et al.*, 2008*b*). The angles between the mean plane of the prop-2-ene-1-one group and the mean planes of the 3-chlorophenyl and 4-chlorophenyl rings are 24.1 (2)° and 29.63°, respectively. While no classical hydrogen bonds are present, weak intermolecular C–H···π-ring interactions are observed which contribute to the stability of crystal packing (Table 1).

### Experimental

50% KOH was added to a mixture of 3-chloroacetophenone (0.01 mol) and *p*-chlorobenzaldehyde (0.01 mol) in 25 ml of ethanol. The mixture was stirred for an hour at room temperature and the precipitate was collected by filtration and purified by recrystallization from ethanol: yield 70% . Single crystals (m.p. 406–408 K) were grown from ethyl acetate by the slow evaporation method. Anal. found: C, 64.96; H, 3.61%; calc. for C_15_H_10_Cl_2_O: C 65.01; H, 3.64%.

### Refinement

All of the H atoms were placed in their calculated positions and then refined using the riding model with C—H = 0.95 Å, and with *U*_iso_(H) = 1.17–1.24*U*_eq_(C).

### Crystal data

**Table:**

C_15_H_10_Cl_2_O	*Z* = 2
*M**_r_* = 277.13	*F*(000) = 284
Triclinic, *P*1	*D*_x_ = 1.499 Mg m^−^^3^
Hall symbol: -P 1	Melting point = 406–408 K
*a* = 5.8884 (9) Å	Cu *K*α radiation, λ = 1.54184 Å
*b* = 7.3328 (9) Å	Cell parameters from 2900 reflections
*c* = 14.6752 (16) Å	θ = 6.2–73.9°
α = 102.821 (10)°	µ = 4.61 mm^−^^1^
β = 95.003 (10)°	*T* = 110 K
γ = 92.933 (11)°	Block, colorless
*V* = 613.88 (14) Å^3^	0.53 × 0.33 × 0.28 mm

### Data collection

**Table:**

Oxford Diffraction Gemini R CCD diffractometer	2402 independent reflections
Radiation source: Enhance (Cu) X-ray Source	2147 reflections with *I* > 2σ(*I*)
graphite	*R*_int_ = 0.035
Detector resolution: 10.5081 pixels mm^-1^	θ_max_ = 73.9°, θ_min_ = 6.2°
ω scans	*h* = −7→7
Absorption correction: multi-scan (*CrysAlis PRO*; Oxford Diffraction, 2007)	*k* = −4→9
*T*_min_ = 0.067, *T*_max_ = 0.275	*l* = −18→18
4041 measured reflections	

### Refinement

**Table:**

Refinement on *F*^2^	Primary atom site location: structure-invariant direct methods
Least-squares matrix: full	Secondary atom site location: difference Fourier map
*R*[*F*^2^ > 2σ(*F*^2^)] = 0.047	Hydrogen site location: inferred from neighbouring sites
*wR*(*F*^2^) = 0.133	H-atom parameters constrained
*S* = 1.03	*w* = 1/[σ^2^(*F*_o_^2^) + (0.0992*P*)^2^ + 0.1931*P*] where *P* = (*F*_o_^2^ + 2*F*_c_^2^)/3
2402 reflections	(Δ/σ)_max_ < 0.001
163 parameters	Δρ_max_ = 0.48 e Å^−^^3^
0 restraints	Δρ_min_ = −0.38 e Å^−^^3^

### Special details

**Table:**

Geometry. All e.s.d.'s (except the e.s.d. in the dihedral angle between two l.s. planes) are estimated using the full covariance matrix. The cell e.s.d.'s are taken into account individually in the estimation of e.s.d.'s in distances, angles and torsion angles; correlations between e.s.d.'s in cell parameters are only used when they are defined by crystal symmetry. An approximate (isotropic) treatment of cell e.s.d.'s is used for estimating e.s.d.'s involving l.s. planes.
Refinement. Refinement of *F*^2^ against ALL reflections. The weighted *R*-factor *wR* and goodness of fit *S* are based on *F*^2^, conventional *R*-factors *R* are based on *F*, with *F* set to zero for negative *F*^2^. The threshold expression of *F*^2^ > σ(*F*^2^) is used only for calculating *R*-factors(gt) *etc*. and is not relevant to the choice of reflections for refinement. *R*-factors based on *F*^2^ are statistically about twice as large as those based on *F*, and *R*- factors based on ALL data will be even larger.

### Fractional atomic coordinates and isotropic or equivalent isotropic displacement parameters (Å^2^)

**Table:**

	*x*	*y*	*z*	*U*_iso_*/*U*_eq_	
Cl1	0.57079 (8)	−0.05763 (7)	0.10784 (3)	0.0235 (2)	
Cl2	−0.09011 (9)	0.72931 (8)	0.93921 (3)	0.0286 (2)	
O1	0.7088 (2)	0.2250 (2)	0.47821 (10)	0.0233 (4)	
C1	0.3892 (3)	0.1004 (3)	0.36950 (14)	0.0161 (4)	
C2	0.5165 (3)	0.0748 (3)	0.29158 (14)	0.0154 (4)	
H2A	0.6696	0.1269	0.2980	0.019*	
C3	0.4159 (3)	−0.0275 (3)	0.20525 (14)	0.0164 (4)	
C4	0.1936 (4)	−0.1087 (3)	0.19394 (15)	0.0203 (4)	
H4A	0.1276	−0.1790	0.1343	0.024*	
C5	0.0704 (3)	−0.0849 (3)	0.27143 (15)	0.0198 (4)	
H5A	−0.0810	−0.1409	0.2649	0.024*	
C6	0.1652 (3)	0.0201 (3)	0.35873 (14)	0.0179 (4)	
H6A	0.0777	0.0371	0.4110	0.021*	
C7	0.5008 (3)	0.2095 (3)	0.46235 (14)	0.0177 (4)	
C8	0.3516 (3)	0.2980 (3)	0.53315 (14)	0.0191 (4)	
H8A	0.1959	0.3100	0.5139	0.023*	
C9	0.4301 (3)	0.3613 (3)	0.62341 (14)	0.0169 (4)	
H9A	0.5857	0.3439	0.6403	0.020*	
C10	0.3008 (3)	0.4551 (3)	0.69935 (14)	0.0161 (4)	
C11	0.3922 (3)	0.4779 (3)	0.79294 (14)	0.0174 (4)	
H11A	0.5390	0.4357	0.8058	0.021*	
C12	0.2738 (3)	0.5603 (3)	0.86707 (14)	0.0202 (4)	
H12A	0.3365	0.5729	0.9302	0.024*	
C13	0.0614 (4)	0.6243 (3)	0.84698 (14)	0.0189 (4)	
C14	−0.0325 (3)	0.6083 (3)	0.75532 (14)	0.0177 (4)	
H14A	−0.1765	0.6555	0.7430	0.021*	
C15	0.0860 (3)	0.5226 (3)	0.68181 (14)	0.0167 (4)	
H15A	0.0213	0.5094	0.6189	0.020*	

### Atomic displacement parameters (Å^2^)

**Table:**

	*U*^11^	*U*^22^	*U*^33^	*U*^12^	*U*^13^	*U*^23^
Cl1	0.0286 (3)	0.0275 (3)	0.0145 (3)	0.0029 (2)	0.0075 (2)	0.0028 (2)
Cl2	0.0267 (3)	0.0389 (4)	0.0174 (3)	0.0087 (2)	0.0064 (2)	−0.0026 (2)
O1	0.0188 (7)	0.0308 (9)	0.0191 (7)	0.0014 (6)	0.0024 (6)	0.0028 (6)
C1	0.0191 (9)	0.0144 (9)	0.0156 (10)	0.0038 (7)	0.0033 (7)	0.0041 (7)
C2	0.0154 (9)	0.0139 (9)	0.0177 (10)	0.0025 (7)	0.0025 (7)	0.0045 (7)
C3	0.0193 (10)	0.0154 (9)	0.0154 (9)	0.0045 (7)	0.0053 (7)	0.0035 (7)
C4	0.0231 (10)	0.0164 (10)	0.0196 (10)	0.0005 (8)	−0.0013 (8)	0.0017 (8)
C5	0.0162 (9)	0.0168 (10)	0.0264 (11)	−0.0002 (8)	0.0005 (8)	0.0063 (8)
C6	0.0169 (9)	0.0189 (10)	0.0201 (10)	0.0038 (8)	0.0066 (7)	0.0067 (8)
C7	0.0205 (10)	0.0182 (10)	0.0160 (10)	0.0031 (8)	0.0048 (7)	0.0055 (8)
C8	0.0193 (10)	0.0214 (10)	0.0167 (10)	0.0041 (8)	0.0049 (7)	0.0029 (8)
C9	0.0181 (9)	0.0142 (9)	0.0194 (10)	0.0007 (7)	0.0053 (7)	0.0047 (8)
C10	0.0182 (10)	0.0132 (9)	0.0168 (10)	−0.0016 (7)	0.0038 (7)	0.0031 (7)
C11	0.0183 (10)	0.0147 (10)	0.0183 (10)	−0.0002 (7)	0.0010 (7)	0.0026 (7)
C12	0.0230 (10)	0.0213 (10)	0.0148 (9)	0.0004 (8)	0.0007 (7)	0.0015 (8)
C13	0.0216 (10)	0.0174 (10)	0.0167 (10)	−0.0002 (8)	0.0067 (8)	0.0006 (7)
C14	0.0169 (9)	0.0150 (10)	0.0211 (10)	0.0006 (7)	0.0033 (7)	0.0036 (8)
C15	0.0194 (10)	0.0162 (10)	0.0142 (9)	−0.0004 (8)	0.0014 (7)	0.0034 (7)

### Geometric parameters (Å, °)

**Table:**

Cl1—C3	1.7414 (19)	C8—C9	1.335 (3)
Cl2—C13	1.743 (2)	C8—H8A	0.9500
O1—C7	1.221 (2)	C9—C10	1.469 (3)
C1—C6	1.398 (3)	C9—H9A	0.9500
C1—C2	1.405 (3)	C10—C11	1.401 (3)
C1—C7	1.495 (3)	C10—C15	1.405 (3)
C2—C3	1.386 (3)	C11—C12	1.385 (3)
C2—H2A	0.9500	C11—H11A	0.9500
C3—C4	1.391 (3)	C12—C13	1.389 (3)
C4—C5	1.385 (3)	C12—H12A	0.9500
C4—H4A	0.9500	C13—C14	1.386 (3)
C5—C6	1.393 (3)	C14—C15	1.386 (3)
C5—H5A	0.9500	C14—H14A	0.9500
C6—H6A	0.9500	C15—H15A	0.9500
C7—C8	1.480 (3)		
			
C6—C1—C2	119.42 (18)	C7—C8—H8A	119.1
C6—C1—C7	122.00 (17)	C8—C9—C10	126.68 (19)
C2—C1—C7	118.57 (17)	C8—C9—H9A	116.7
C3—C2—C1	119.19 (17)	C10—C9—H9A	116.7
C3—C2—H2A	120.4	C11—C10—C15	118.27 (19)
C1—C2—H2A	120.4	C11—C10—C9	119.40 (18)
C2—C3—C4	121.81 (18)	C15—C10—C9	122.33 (18)
C2—C3—Cl1	119.60 (15)	C12—C11—C10	121.58 (19)
C4—C3—Cl1	118.59 (16)	C12—C11—H11A	119.2
C5—C4—C3	118.61 (19)	C10—C11—H11A	119.2
C5—C4—H4A	120.7	C11—C12—C13	118.49 (19)
C3—C4—H4A	120.7	C11—C12—H12A	120.8
C4—C5—C6	120.93 (18)	C13—C12—H12A	120.8
C4—C5—H5A	119.5	C14—C13—C12	121.62 (19)
C6—C5—H5A	119.5	C14—C13—Cl2	119.12 (16)
C5—C6—C1	120.02 (18)	C12—C13—Cl2	119.25 (16)
C5—C6—H6A	120.0	C15—C14—C13	119.30 (19)
C1—C6—H6A	120.0	C15—C14—H14A	120.4
O1—C7—C8	121.66 (19)	C13—C14—H14A	120.4
O1—C7—C1	120.42 (18)	C14—C15—C10	120.71 (18)
C8—C7—C1	117.93 (17)	C14—C15—H15A	119.6
C9—C8—C7	121.72 (19)	C10—C15—H15A	119.6
C9—C8—H8A	119.1		
			
C6—C1—C2—C3	1.0 (3)	C1—C7—C8—C9	164.85 (19)
C7—C1—C2—C3	179.45 (17)	C7—C8—C9—C10	178.46 (18)
C1—C2—C3—C4	−1.3 (3)	C8—C9—C10—C11	166.7 (2)
C1—C2—C3—Cl1	179.19 (14)	C8—C9—C10—C15	−12.9 (3)
C2—C3—C4—C5	0.4 (3)	C15—C10—C11—C12	1.6 (3)
Cl1—C3—C4—C5	179.94 (15)	C9—C10—C11—C12	−178.04 (17)
C3—C4—C5—C6	0.8 (3)	C10—C11—C12—C13	−1.2 (3)
C4—C5—C6—C1	−1.1 (3)	C11—C12—C13—C14	−0.4 (3)
C2—C1—C6—C5	0.2 (3)	C11—C12—C13—Cl2	−179.77 (15)
C7—C1—C6—C5	−178.20 (18)	C12—C13—C14—C15	1.4 (3)
C6—C1—C7—O1	155.76 (19)	Cl2—C13—C14—C15	−179.14 (14)
C2—C1—C7—O1	−22.7 (3)	C13—C14—C15—C10	−1.0 (3)
C6—C1—C7—C8	−24.7 (3)	C11—C10—C15—C14	−0.4 (3)
C2—C1—C7—C8	156.89 (18)	C9—C10—C15—C14	179.15 (17)
O1—C7—C8—C9	−15.6 (3)		

### Hydrogen-bond geometry (Å, °)

**Table:**

*D*—H···*A*	*D*—H	H···*A*	*D*···*A*	*D*—H···*A*
C2—H2A···Cg2^i^	0.95	2.98	3.608 (2)	125
C5—H5A···Cg2^ii^	0.95	2.88	3.488 (2)	126
C14—H14A···Cg1^iii^	0.95	2.77	3.474 (2)	131

Symmetry codes: (i) −*x*+1, −*y*+1, −*z*+1; (ii) −*x*, −*y*, −*z*+1; (iii) −*x*, −*y*+1, −*z*+1.
